# Relationship between retention intention, perceived organizational support, and medical narrative ability among nurses: a cross-sectional multi-center study

**DOI:** 10.3389/frhs.2025.1648464

**Published:** 2025-08-15

**Authors:** Yanjia Li, Rong Zhang, Limei Kang, Guoting Ma

**Affiliations:** ^1^ Department of Emergency, Sichuan Taikang Hospital, Chengdu, Sichuan, China; ^2^ Department of Nursing, Sichuan Taikang Hospital, Chengdu, Sichuan, China; ^3^ Department of Anesthesiology, Gansu Provincial Hospital, Lanzhou, Gansu, China

**Keywords:** nurse, retention intention, perceived organizational support, medical narrative ability, cross-sectional multi center

## Abstract

**Background:**

MNA is widely regarded as an important factor in promoting the construction of humanistic hospitals. Nurses are the medical professionals who have the highest participation in the clinical practice of narrative medicine and humanistic nursing in hospitals. Therefore, it is crucial to study the impact of nurses’ RI and POS on MNA.

**Objectives:**

This study aims to determine the effects of nurses’ perceived organizational support (POS) and retention intention (RI) on nurses’ medical narrative ability (MNA) and to reveal the mediating role of RI in the possible impact.

**Methods:**

A cross-sectional descriptive correlational study was performed. Survey data were gathered from 1,831 practicing nurses working in eight tertiary general hospitals across China. The questionnaire was divided into four sections: a sociodemographic characteristics survey, a medical narrative ability scale, a perceived organizational support scale, and a Chinese questionnaire for nurses’ intention to remain employed. Data analysis was conducted using SPSS 27.0.

**Results:**

The survey results show that the relationships among RI, POS, and MNA are all significant, with correlation coefficients ranging from 0.344 to 0.468. The indirect effect of POS on MNA is 1.267, accounting for 29.12% of the cumulative effect.

**Conclusion:**

This study reveals that nurses’ POS not only directly enhances their MNA but also indirectly has a profound impact on this ability through the mediating mechanism of the RI. This finding indicates that nursing managers both domestically and internationally should pay close attention to nurses’ perception levels of organizational support and their RI, and formulate precise and effective intervention strategies, thereby using this as a potential organizational management approach to enhance nurses’ MNA.

## 1 Introduction

With the progress of science and technology, new methods and technologies have been provided for diagnosing and treating diseases. However, medicine is increasingly inclined to silent technology. It begins to “emphasize technology over humanity”, resulting in the estrangement between medicine and humanity, resulting in more and more doctor-patient conflicts or medical disputes (1). Against this background, the term of “Narrative Medicine (NM)” was first proposed by the American scholar Rita Charon (2). NM refers to medical practice carried out by medical staff through their possession of narrative ability, and “narrative ability” specifically refers to the ability to perceive, internalize, interpret, and generate empathy for disease stories. NM is a new tool for human beings to reproduce and understand the potential of pain and disease (3). At the same time, it listens to and sees patients alienated by science and technology, views diseases, patients as disease carriers, and the stories behind patients from another perspective (4). It is also a critical intervention method to bring medical humanities into clinical practice (5). However, NM was introduced to China relatively late. In the process of Sinicization, in order to distinguish it from the narrative ability in literature, domestic scholars mostly express it in terms of Medical Narrative Ability (MNA). Nurses are among the essential participants in the healthcare service industry and the medical group with the most contact with patients. They have made significant contributions to the quality of healthcare service and are also one of the influential practitioners of the clinical practice of NM (6). Wang et al. (7) pointed out that nurses' MNA can provide nurses with an opportunity to understand patients' illness experience, narrow the emotional distance between nurses and patients, and provide patients with the highest spiritual care, which is conducive to helping patients' psychological relief and spiritual comfort, improving patients' prognosis, and promoting the benign development of the nursion-patient relationship. In addition, the improvement of MNA also contributes to the improvement of nurses' empathy ability, professionalism, affinity and self-behavior reflection (8, 9). Due to the fact that the localization development of NM in clinical practice in China still faces dual challenges in theory and application, nursing staff generally exhibit a structural deficiency in MNA. It is not only difficult for them to accurately identify implicit demands such as emotional fluctuations and value conflicts of patients during the treatment process through disease narratives, but they also lack systematic training to transform medical humanistic theories into clinical communication skills (10). Therefore, how to construct a culturally responsive NM training system and enhance nurses' NM capabilities is not only a key approach to achieving the transformation of the “technology—humanities” dual-track equal emphasis nursing model, but also an important cultural medium to promote the implementation of the doctor-patient joint decision-making model.

Retention Intention (RI) means that employees do not intend to leave and will not try to find new jobs (11). It is an essential indicator for evaluating the stability of the nursing team (12). Given that the current shortage of nurses will continue to worsen (13), although nurses have not yet quit, the negative emotions brought by a low willingness to stay will still reduce their work efficiency and quality, produce job burnout, reduce their subjective well-being, damage the physical and mental health of patients and nurses themselves, and also affect the health care organizations (14, 15). It even has a more significant impact on the country and the international community. Kim and Yang (16) pointed out that the enhancement of nurses' RI is positively correlated with their degree of love for work, self-learning ability, clarity of career planning, patience and responsibility, as well as the ability to pay attention to the physical and mental conditions of patients. Furthermore, a stronger RI can also promote the harmony of nurse-patient communication and enhance the level of humanistic care in nursing work (17). Therefore, we speculated that nurses' RI may also affect their MNA.

Perceived Organizational Support (POS), as the core construct of organizational support theory, specifically refers to the overall psychological perception of employees that the organization values their work contributions and cares about their well-being needs (18). Previous studies have shown that enhancing employees' POS can not only effectively stimulate nurses' work enthusiasm and sense of belonging, but also strengthen their sense of collective honor and professional achievement, thereby further promoting team cohesion and work quality (19). This mechanism prompts nurses to actively give back their own value to the organization, continuously improve their professional skills, and fully commit themselves to providing patients with better nursing services. In addition, some scholars pointed out that a good POS can build a harmonious department atmosphere, nurture patient relationships, and improve nurses' humanistic caring ability (20). MNA is also the key to promoting nurses' humanistic nursing work (9). With the development of the global population ageing, global However, several studies consistently show that nurses often receive the most minor organizational support in medical institutions (21), and this phenomenon is more evident in China. The International Council of Nurses (ICN) highlighted in 2024 that recurrent financial crises have resulted in constraints on healthcare budgets, and these constraints frequently entail reductions in care services. Despite nursing services being the cornerstone of the healthcare system, they have persistently encountered funding shortages and societal under appreciation of their significance (22). Therefore, exploring whether the POS can affect nurses' MNA is particularly important.

According to the principle of reciprocity in Social Exchange Theory (SET), when one party generously extends a helping hand, offers assistance and support to the other party, the recipient should assume the corresponding responsibility to reciprocate and demonstrate reciprocal kindness and respect through actions (23). According to this theory, when nurses receive adequate care and support from the organization, their POS will be significantly enhanced, thereby stimulating the sense of responsibility and positive attitude deep within them, and having a profound positive impact on work engagement and performance, further strengthening their RI (24).The research of Ho et al. (25) also indicates that the POS of nurses can effectively shape their RI, reduce turnover rates, curb the loss of nursing talent, and thereby maintain the stability and cohesion of the nursing team. Furthermore, the broaden-and-build theory further explains that positive emotional experiences can not only broaden an individual's cognitive boundaries and behavioral potential but also deepen the reserves of resources such as physical strength, intelligence, and social coordination ability, continuously injecting momentum into personal growth and development, and comprehensively enhancing overall quality (26).When the POS, as a positive emotional experience, can enhance nurses' RI and their professional identity, make them full of compassion for patients and have enough patience to pay attention to patients' physical, psychological, social and spiritual problems, nurses' empathy and reflective ability will also be improved, and their MNA may also be affected.

Following social exchange theory and extension-construction theory, we consider POS as a positive emotional experience, RI as an individual response, and MNA as an outcome indicator. Therefore, this study aims to explore in depth the relationship between nurses' RI, cardiac POS, and MNA, and further analyze their potential mediating mechanisms. The results of this study will provide a solid theoretical basis and innovative evidence for constructing a comprehensive program that can effectively improve nurses' MNA. Therefore, this study proposes two research hypotheses:

Hypothesis 1: POS, RI, and MNA are positively correlated.
Hypothesis 2: It is assumed that RI plays a mediating role in the relationship between POS and MNA.

## 2 Methods

### 2.1 Design and participants

This study adopted a descriptive cross-sectional design and used convenience sampling to conduct an online questionnaire survey among qualified practicing nurses in eight tertiary hospitals across the country from April to June 2024. The inclusion criteria were as follows: (i) aged 18 years or older; (ii) having worked at the current institution for at least six months; (iii) voluntarily consenting to participate in this research. The exclusion criteria encompassed the following scenarios: (i) nurses are either on leave or participating in off-site training programs; (ii) nurses who withdrew from the study during its course.

### 2.2 Instrumentation

This study used four measures to collect data: A questionnaire for the general characteristics of the participants, the Medical Narrative Ability Scale (MNAS), the Perceived Organizational Support Scale (POSS), and the Chinese Questionnaire for Nurse Intention to Remain Employed (C-QNIRE).

Nurses' MNA was assessed using the MNAS, a tool developed by Ma et al. (27). The MNAS comprises 27 items divided into three domains: understanding and response, pay attention and listen, reflection and representation. Each item is rated on a 7-point Likert scale, ranging from 1 (“completely inconsistent”) to 7 (“completely consistent”). The total score of this scale ranges from 27 to 189 points, with higher scores indicating higher levels of MNA. In order to better present the results, the original authors of the MNAS divided their results into three levels based on the total score of the scale: low (27–144), medium (145–163), and high (164–189). In this study, the Cronbach's alpha coefficient for the MNAS was 0.967 and the content validity was 0.890.

The POSS scale developed by Zuo and Yang (28) was adopted to evaluate the level of POS among nurses. This questionnaire consists of 13 elaborately designed items, covering two core areas: emotional support and instrumental support. Each item is quantitatively evaluated based on the Likert 5-point scoring system, with the scoring range from 1 (“completely inapplicable”) to 5 (“completely applicable”), thereby comprehensively reflecting the actual feelings of the test-takers. The total score of this scale ranges from 13 to 65 points, with higher scores indicating higher levels of POS. In order to better present the results, the original authors of the POSS divided their results into three levels based on the total score of the scale: low (13–27), medium (28–50), and high (51–65). In this study, the Cronbach's alpha coefficient for the POSS was 0.981 and the content validity was 0.880.

The RI of nurses was quantitatively evaluated through the C-QNIRE, which was translated and introduced by Tao and Wang (29).The C-QNIRE scale consists of items in six dimensions. Each item is meticulously evaluated based on the Likert 5-point scoring method, with the score range from 1 to 5, comprehensively reflecting the psychological tendencies of nurses in career choices. The total score of this scale ranges from 6 to 30 points, with higher scores indicating higher levels of RI. In order to better present the results, the original authors of the C-QNIRE divided their results into three levels based on the total score of the scale: low (6–13), medium (14–23), and high (25–30). In this study, the Cronbach's alpha coefficient of the C-QNIRE was 0.799, and the retest Cronbach's alpha coefficient was 0.749.The content validity correlation coefficients of the items in C-QNIRE range from 0.590 to 0.810.

### 2.3 Ethical consideration and data collection

The Ethics committee of Sichuan Taikang Hospital provided ethical permission for the study. The lead researchers contacted the heads of eight hospitals to ask for approval for the data collection. After obtaining permission to conduct research, researchers will inform the purpose of the questionnaire, the filling time (15–20 min) and other precautions through the social software WeChat and obtain the informed consent of participants. The survey data was collected anonymously through an online platform called “So Jump”, and all data will be kept strictly confidential. The researchers will then send an online questionnaire via WeChat. All questions are required. If participants do not complete all the contents of the questionnaire, they cannot submit it. In addition, the exclusion criteria for “invalid” questionnaires should include: (I) Lack of regular answering patterns, such as choosing the same option for more than 10 consecutive questions; (II) Completion time less than 10 min; (III) Excessive missing values, such as the proportion of missing values for core variables being ≥20%. Any questionnaire meeting any of these criteria is considered invalid.

### 2.4 Data analysis

Data processing and analysis were accomplished using SPSS 27.0 software. A normality test was conducted before data analysis. All data were subjected to detailed descriptive statistical analysis through percentages, means, and standard deviations to present the basic characteristics of the data. The relationship between variables was explored in depth with the aid of Pearson correlation analysis. One-way analysis was performed using the independent samples *t*-test and one-way analysis of variance (ANOVA). Furthermore, based on Model 4 of Hayes' PROCESS macro and combined with the bootstrapping method, the mediating effect of RI in the relationship between POS and MNA was evaluated. The significance test of the mediating effect was conducted using the bootstrapping method with a 95% bias-corrected CI, and the two-sided test with *p* < 0.05 was used as the criterion for judging statistical significance.

## 3 Results

### 3.1 Participant characteristics and single factor analysis of MNA

A total of 1,840 questionnaires were collected, though nine were valid after excluding the unqualified ones, with an effective rate of 99.51%. A total of 1,831 subjects were included in this study. Among them, the proportion of females was as high as 97.4%, while that of males was only 2.6%. It is worth noting that only a few participants did not obtain a bachelor's degree or above, and this proportion was 28.6%. Furthermore, in terms of work experience, the proportion of the group with less than 5 years of work experience was 29%. More strikingly, the proportion of subjects working fewer than 40 h per week was only 10.6%, indicating a relatively high work intensity characteristic. The single factor analysis results of MNA are detailed in Table 1.

**Table 1:** Comparison of MNA among nurses with different sociodemographic characteristics (*n* = 1,831).

Variables	*N* (%)	Mean ± SD	*t*/F	*p*
Gender			−0.505	0.614
Female	1,783 (97.4)	154.43 ± 22.95		
male	48 (2.6)	156.13 ± 22.52		
Age (years)			2.433	0.033
18–25	210 (11.5)	151.57 ± 19.93		
26–30	601 (32.8)	154.01 ± 23.90		
31–35	500 (27.3)	154.11 ± 23.04		
36–40	259 (14.1)	154.52 ± 21.90		
41–45	124 (6.8)	158.06 ± 21.39		
46–60	137 (7.5)	159.00 ± 25.03		
Marital status			5.403	0.005
Married	1,348 (73.6)	154.89 ± 23.33		
Single	410 (22.4)	152.00 ± 21.92		
Divorce or others	73 (4.0)	160.79 ± 19.40		
Education level			1.190	0.304
High school	12 (0.7)	163.83 ± 17.45		
College	511 (27.9)	154.94 ± 22.52		
University or above	1,308 (71.4)	154.21 ± 23.13		
Professional title			0.317	0.813
nurse	267 (14.6)	154.03 ± 21.98		
senior nurse	7,389 (40.3)	154.00 ± 23.21		
nurse-in-charge	701 (38.3)	155.10 ± 22.54		
associate chief nurse or above	125 (6.8)	154.70 ± 25.49		
Department			5.528	<0.001
Internal medicine	688 (37.6)	151.17 ± 23.42		
Surgical	432 (23.6)	155.86 ± 22.95		
Pediatric	86 (4.7)	159.34 ± 19.31		
Obstetrics and gynecology	115 (6.3)	158.16 ± 22.11		
Outpatient	83 (4.5)	162.37 ± 17.29		
Emergency	87 (4.8)	155.10 ± 19.19		
Others	340 (18.6)	154.84 ± 24.14		
Working experience (years)			4.033	0.003
<3	212 (11.6)	149.91 ± 22.22		
3–5	319 (17.4)	152.88 ± 23.16		
6–10	426 (23.3)	154.33 ± 22.08		
11–15	521 (28.5)	155.63 ± 23.25		
>15	353 (19.3)	157.14 ± 23.27		
Income (RMB/month)			2.268	0.079
<3,000	106 (5.8)	149.52 ± 22.08		
3,000–5,000	699 (38.2)	153.95 ± 24.49		
5,001–10,000	978 (53.4)	155.35 ± 21.79		
>10,000	48 (2.6)	155.31 ± 23.07		
Narrative medicine training			−8.219	<0.001
No	908 (49.6)	150.11 ± 23.43		
Yes	923 (50.4)	158.77 ± 21.60		
Social activities			−4.795	<0.001
No	757 (41.3)	151.35 ± 24.95		
Yes	1,074 (58.7)	156.68 ± 21.13		
Working week (hours)			2.574	0.054
<40	195 (10.6)	154.27 ± 22.83		
40–45	1,038 (56.7)	155.69 ± 22.47		
46–50	402 (22.0)	152.50 ± 22.55		
>50	196 (10.7）	152.33 ± 25.75		

### 3.2 Correlation between nurses’ RI, POS and MNA

The scores for MNA, RI and POS were 154.48 ± 22.93, 22.79 ± 3.71, and 46.68 ± 11.00, respectively. Moreover, the findings from the Pearson correlation analysis revealed a significant positive association between MNA and both RI (*r* = 0.344, *p* < 0.01) and POS (*r* = 0.348, *p* < 0.01). Notably, a robust positive correlation was also observed between RI and POS (*r* = 0.468, *p* < 0.01) (Table 2).

**Table 2:** Scores and correlation coefficients of the study variables (*n* = 1,831).

Variables	M ± SD	1	2	3
1. MNA	154.48 ± 22.93	1	–	–
2. RI	22.79 ± 3.71	0.344**	1	–
3. POS	46.68 ± 11.00	0.348**	0.468**	1

** *p* *<* *0.01.*

### 3.3 Mediating effect of nurse's retention intention, POS and MNA

**Figure 1:**
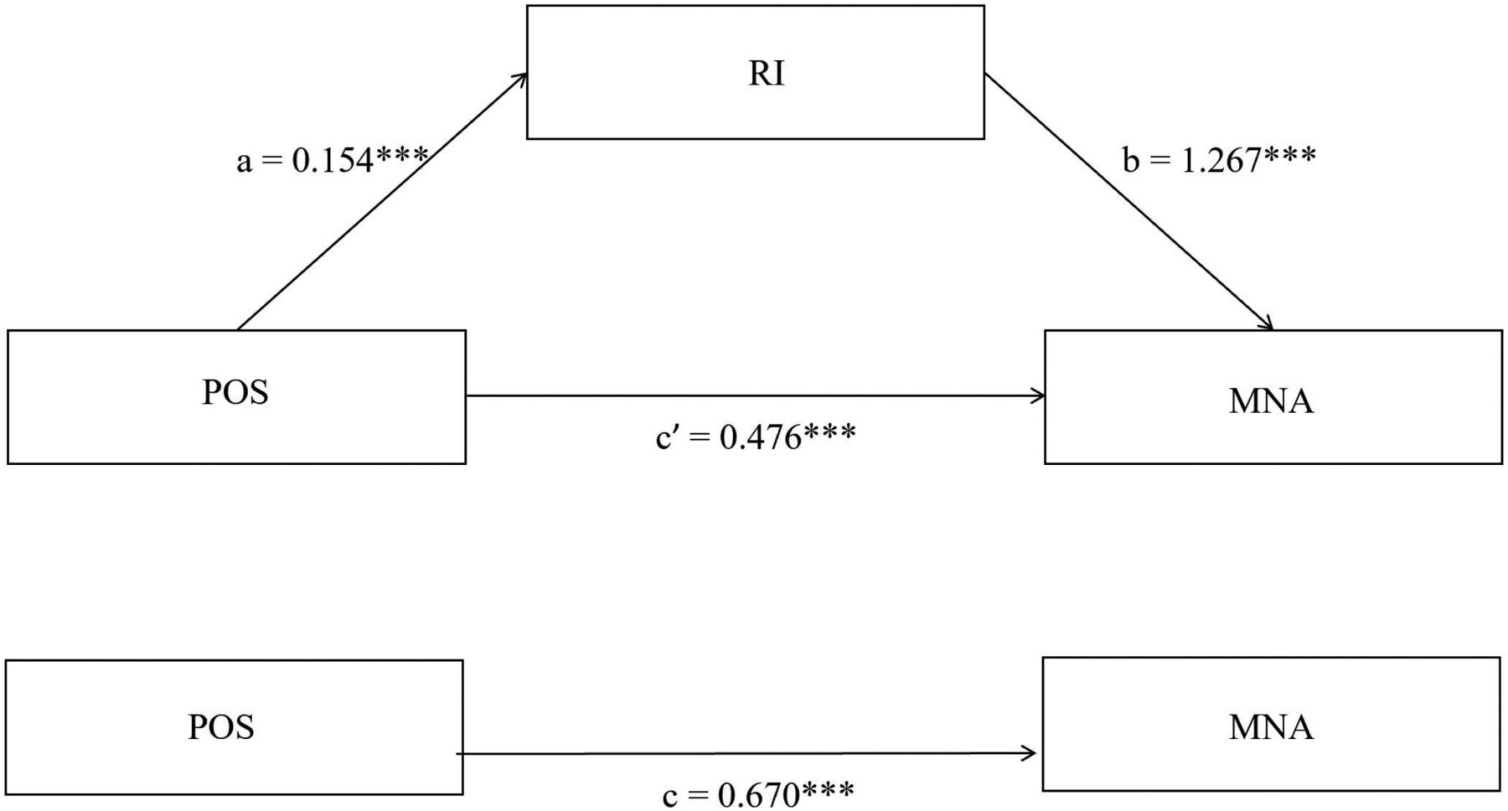
Mediation model of RI on the relationship between POS and MNA of nurses. *** *p* < 0.001.

Based on Hayes' PROCESS macroscopic Model 4, analyze the mediating role of RI between POS and MNA. The Control variables are the meaningful single factor analysis of MNA. The findings revealed that POS was a significant predictor of RI (*a* = 0.154, SE = 0.007, *p* < 0.001). Additionally, RI was identified as a substantial predictor of MNA (*b* = 1.267, SE = 0.154, *p* < 0.001). POS also had a direct influence on MNA (c’ = 0.476, SE = 0.051, *p* < 0.001). The bias-corrected percentile Bootstrap method demonstrated that the 95% confidence interval did not encompass zero, indicating that RI partially mediated the link between POS and MNA. The mediation effect constituted 29.12% of the overall effect (Table 3; Figure 1).

**Table 3:** Mediating effect of RI between POS and MNA (*n* = 1,831).

Effect	Path	*β*	Bootstrap 95% CI	*SE*	*t*	*p*
Direct effect	POS → MNA	0.476 (c’)	0.376, 0.575	0.051	9.382	<0.001
Indirect effect	POS → RI	0.154 (a)	0.140, 0.167	0.007	22.512	<0.001
	RI → MNA	1.267 (b)	0.965, 1.569	0.154	8.222	<0.001
Total effect	POS → MNA	0.670 (c)	0.580, 0.760	0.046	14.676	<0.001

SE, standard error; CI, confidence interval.

## 4 Discussion

This study explored the relationship between POS, RI, and MNA of nurses, as well as the possible mediating role of RI between POS and MNA. First, in this study, the total average score of nurses’ MNA is at a medium level, similar to the results of (27). This may be because the subjects investigated in this study all work in tertiary general hospitals, with many patients in tertiary hospitals, severe imbalance in the nursery-patient ratio, and nurses' excellent work pressure and heavy tasks (30). Nurses lack the time and energy to listen to patients' demands (31) and understand and respond to patients' narratives, and their own MNA will also be affected. Secondly, compared with the study of Daryazadeh et al. (32) abroad, nurses in this study scored lower in MNA. Due to the earlier development of narrative medicine abroad, nurses had more clinical practice experience. However, NM in China is still in the initial stage of development, and most nurses have not received systematic theoretical and practical training on NM (33), which also suggests that nursing managers and educators should pay attention to the cultivation of nurses' MNA.

The results of this study indicate that nurses' RI is a positive predictor of MNA. According to the results, hypothesis 1 is confirmed. The higher the RI of nurses, the higher the level of their MNA. It is of great significance to understand nurses' RI in time and improve their retention rate to alleviate the shortage of nurses (34). Min et al. (11) Showed that when nurses have firm RI, they have positive self-orientation, precise career planning, are full of hope for their career prospects, affirm their social status, control their emotions well, adopt a positive attitude to cope with difficulties, and show stronger stress resistance and resilience in difficult situations. At the same time, nurses will be more focused on their work, get along harmoniously with patients, and improve their empathy and reflection ability (35). Their MNA will also be affected by the process of continuous practice. Therefore, hospital and nursing managers should attach great importance to the nurses' RI and take the initiative to intervene to increase nurses' RI to improve nurses' MNA.

In our study, we also found that nurses’ POS was also a positive predictor of MNA. According to the results, hypothesis 2 is confirmed. The stronger the POS, the higher the level of MNA of nurses. When nurses find that they get roughly the same organizational support and input in their career, their psychology will be relatively balanced, and when their organizations provide more emotional and instrumental support, nurses will have a stronger POS, which makes it easy to have a sense of security and trust. They will work hard to return to the organization (36). Based on the humanistic nursing theory (37), the care of the organization for nurses can profoundly shape the quality of nurse-patient interaction. When nurses truly feel the warmth and support from the organization, they are more inclined to turn this kindness into actions, convey warmth and care to patients, and thereby cultivate positive professional values. Beyond the treatment, they will also proactively provide narrative support to patients to help them build psychological resilience, thereby effectively enhancing the MNA of nurses. Therefore, it is suggested that nursing managers attach great importance to the POS of nurses. Through emotional comfort and tool empowerment, they should provide all-round support and assistance to nurses to stimulate their inner motivation and promote the overall improvement of nursing quality.

Finally, the most crucial finding of this study lies in revealing the partial mediating role of nurses' RI between POS and MNA, thereby verifying Hypothesis 2. Based on the social exchange theory (23), we are able to construct a framework to deeply understand the decision-making mechanism of individuals in social interaction-how they weigh benefits and costs and maximize overall well-being through reciprocal obligations. Therefore, when an organization devotes its efforts to the well-being of nurses and fully recognizes their contributions, nurses will respond with more outstanding job performance, higher commitment, and a series of positive behaviors. At the same time, their RI will also increase significantly. Furthermore, existing studies (38) have further confirmed the close association between nurses' POS and RI. In addition, according to the extension-construction theory (26), positive occupational emotional experience can narrow the psychological distance between practitioners and their occupation, make them gradually love their occupation from exclusion, improve their subjective well-being and satisfaction with their occupation, and thus enhance their intention to stay, promote personal growth and development, and improve their ability. The POS is a positive emotional experience (39), which can significantly affect the RI of nurses, and the RI also indirectly adjusts the relationship between the POS and the MNA of nurses. POS provides nurses with emotional support and recognition of their value, fulfilling their basic psychological needs. This sense of being supported and valued significantly enhances nurses' sense of belonging, commitment, and RI. They feel that the organization is a trustworthy and reliable “home”, willing to contribute here for the long term. This stable and positive state of RI creates an environment and motivation for nurses to continuously practice, deeply engage, be willing to try, accumulate knowledge, and concentrate their energy. It is these conditions and mindsets resulting from the RI that enable nurses to have more opportunities, stronger motivation, and better conditions to continuously develop, apply, and improve their MNA. When nurses have a firm RI, they can identify the connotation and significance of narrative medicine, more easily exert their subjective initiative, learn to reflect, patiently listen to patients, timely capture patients' body language and information transmitted by the surrounding environment, find patients' unexpressed spiritual needs in addition to diseases, and provide support and help (40, 41). Nurses' MNA will also improve in clinical practice by focusing on listening, understanding, responding, and empathizing with patients. However, when nurses' RI is low, they are prone to negative emotions (42), which leads to nurses thinking that their working environment is unfair, resulting in reduced job satisfaction, lack of trust and commitment, and their negative attitude will seriously affect nurses' MNA (43, 44). Although RI is one of the important mechanisms for enhancing MNA through POS, it is not the only pathway (for example, POS may also directly influence nurses' MNA by providing relevant training resources or creating a supportive communication atmosphere), which is consistent with the result of our study that RI has a “partial mediating effect” between nurses' POS and MNA.

Therefore, nursing managers should consider the impact of POS on nurses' MNA and recognize the mediating role of RI between POS and MNA. Nursing managers can train nurses with different working years and carry out learning through multiple channels and forms, such as holding lectures and conferences on topics related to narrative medicine, establishing a narrative database (30), facilitating nurses to acquire relevant knowledge of narrative medicine, and encouraging all departments to carry out activities related to narrative medicine to improve nurses' MNA (43–45). Furthermore, nursing managers should also provide nurses with material and emotional support, such as improving the working environment, equipping nurses with more advanced and convenient medical equipment, and offering more opportunities for professional title promotion (46), increasing the welfare benefits of nurses (47), valuing the value of nurses and others (48) to promote the improvement of MNA. Finally, hospital and nursing managers should also focus on factors such as nurses' career development prospects, job matching and humanized management, such as enriching nurses' career development paths and opportunities, reasonable scheduling (49) and carrying out transformational leadership training courses to attract nurses to continue to stay (50) and improve their MNA.

## 5 Limitations

The study focuses on a profound issue closely related to the healthcare industry, namely the relationship among nurses' POS, RI, and MNA. The MNA of nurses significantly impacts the quality of care and the overall operation of medical institutions, thereby highlighting the important value of this study. However, some limitations in the research still need to be noted. Firstly, due to the adoption of a cross-sectional design, the causal relationships among the variables could not be clarified in this study. Therefore, future research can consider further revealing the essential characteristics and potential mechanisms of these relationships through longitudinal investigations or interventional research designs. Secondly, the samples of this study were only derived from eight tertiary hospitals in China, five of which were located in Sichuan Province. This regional concentration limited the universality of the research results to a certain extent. To enhance the reliability of future research conclusions, it is suggested to expand the sample range to cover medical institutions of different levels and regions. Finally, although the measurement tools used have demonstrated excellent reliability and validity in previous studies, data based on self-reports may cause certain response biases. Especially for C-QNIRE, due to constraints such as the research period, sample size, and the goals of the initial validation, we were unable to incorporate a comprehensive structural validity analysis (CFA) at this stage. We will thoroughly explore this issue in future research to make up for this deficiency. Even so, this study, through a theory-driven approach, provides a brand-new perspective for understanding the complex relationship among nurses' POS, RI and MNA.

## 6 Conclusion

This study reveals the mediating role of clinical nurses' RI between their POS and MNA. The POS not only directly enhances nurses' MNA but also indirectly strengthens this ability by enhancing their RI, confirming the key bridging role of the willingness to remain in their positions. In addition, it also provides theoretical basis and important inspirations for optimizing the management of nursing human resources, stabilizing the nursing team, and improving the quality of nursing work.

## Data Availability

The raw data supporting the conclusions of this article will be made available by the authors, without undue reservation.
